# Neoadjuvant chemotherapy in invasive lobular carcinoma of the breast: perspectives based on the survival outcomes

**DOI:** 10.3389/fonc.2025.1655413

**Published:** 2025-09-05

**Authors:** Virginie Gauthier, Anne-Julie Simard, Christine Desbiens, Brigitte Poirier, Dominique Boudreau, Dominique Leblanc, Claudya Morin, Jean-Charles Hogue, Éric Poirier

**Affiliations:** ^1^ Département de chirurgie, CHU de Québec – Université Laval, Quebec City, QC, Canada; ^2^ Faculté de médecine, Université de Laval, Quebec City, QC, Canada; ^3^ Centre des Maladies du Sein, Hôpital du Saint-Sacrement, CHU de Québec – Université Laval, Quebec City, QC, Canada

**Keywords:** breast cancer, invasive lobular carcinoma, neoadjuvant chemotherapy, adjuvant chemotherapy, survival

## Abstract

Whether patients with invasive lobular carcinoma (ILC) can benefit from neoadjuvant chemotherapy (NAC) remains uncertain. In order to attempt to bring some light on the matter, the patients treated for ILC between 1998 and 2016 at a tertiary center specialized in breast diseases were examined according to NAC vs. adjuvant therapy. Among 265 eligible women treated for ILC, 72 received NAC and 193 received adjuvant chemotherapy. In the NAC group, only 4.2% of the patients with ILC achieved a pathological complete response after NAC. Over a mean follow-up of 8 years, after adjusting for confounders (age >55, T-stage, N-stage, surgery type, radiotherapy, and hormonal therapy), the two groups had similar 10-year locoregional recurrence rates (NAC: 90.6%; adjuvant: 93.5%, P=0.110), but the NAC group showed lower 10-year recurrence-free survival (51.8% vs. 72.7%, P=0.0004), 10-year progression-free survival (59.3% vs. 82.0%, P<0.0001), and 10-year overall survival (56.2% vs. 80.7%, P<0.0001). The results suggest that the response of ILC to NAC is poorer than to adjuvant chemotherapy. It is the authors’ opinion that ILC should be considered separately from IDC in clinical trials and guidelines, and that patients with ILC might benefit from a more aggressive surgical approach followed by adjuvant chemotherapy no matter the severity of the disease.

## 1 Introduction

Neoadjuvant chemotherapy (NAC) is a well-established component of breast cancer management (1–3). Although the overall survival (OS) benefit is generally considered similar to that of adjuvant chemotherapy, NAC is mostly used in large, locally advanced breast cancer or in the presence of positive lymph nodes to downstage the disease and allow breast conserving surgery (BCS) (2–6). NAC allows a less aggressive approach to the axillary lymph nodes, potentially sparing an axillary dissection, a procedure associated with high morbidity and complication rates (7). The objective outcome measurement of NAC is the pathological complete response (pCR) (5, 8, 9).

Invasive lobular carcinoma (ILC) is the second most common type of invasive breast cancer after invasive ductal carcinoma (IDC), with a frequency of 4% to 15% (8, 10, 11). ILC differs from IDC in multiple aspects. ILC is more likely to be hormone receptor (HR)-positive compared with IDC and is often mammographically occult (1, 4, 5). ILC is known to lack the adhesion protein E-cadherin, often resulting in larger tumors. It is also associated with higher frequencies of bilateral and multicentric tumors (4, 5, 12). These tumor characteristics often result in ILC being a predictive factor of ineligibility to BCS with a lower pCR rate and higher BCS failure (4–6, 8, 12–17).

The NSABP B-18 trial was the first study to demonstrate a higher rate of BCS in patients with breast cancer receiving NAC (18). That initial study included ILC and IDC. The two types are often included together in clinical trials and the guidelines do not make different recommendations for IDC and ILC despite that many studies suggested major differences in natural history, pathophysiology, and treatment responses between ILC and IDC cancer (4, 5, 12–17).

Previous studies of ILC evaluated the pCR and the possibility of BCS after NAC (6, 8, 9, 14–16), but they did not examine survival. Therefore, this study aimed to evaluate the survival parameters of patients with ILC after NAC compared with adjuvant chemotherapy. Considering that the management of ILC with NAC remains uncertain, the results could help determine the best treatment strategy in patients with ILC.

## 2 Methodological considerations

All patients treated for breast cancer at the “Centre des maladies du sein du CHU de Québec – Université Laval”, a tertiary academic center specialized in breast diseases, are entered in a breast cancer registry since 1976. This retrospective cohort study included patients treated for breast ILC between 1998 and 2016. In this study, 1998 was selected because it is when NAC was started to be used at the study center, and 2016 was selected as the end of the study period to leave a sufficient follow-up for the last patients. At the study center, about 1000 new breast cancer cases are diagnosed and treated each year. The study protocol was approved by the ethics committee of the CHU de Québec – Université Laval. The requirement for individual informed consent was waived due to the retrospective nature of this study.

The inclusion criteria were 1) confirmed histopathological diagnosis of primary pure ILC, 2) completion of all treatments and follow-up at the study center, and 3) patient >18 years of age. Patients with unavailable treatment or follow-up information were excluded. The patients were grouped according to the timing of chemotherapy they received: NAC vs. adjuvant chemotherapy.

The primary outcomes were the OS and the recurrence-free survival (RFS) of patients with ILC receiving NAC vs. adjuvant chemotherapy. The OS was calculated from ILC diagnosis to death. The RFS was calculated from ILC diagnosis to recurrence (biopsy-proven locoregional recurrence or metastases proven radiologically or histologically) or death, whichever occurred first.

The secondary outcomes were the locoregional recurrence (LRR) (i.e., the time from ILC diagnosis to biopsy-proven locoregional recurrence), progression-free survival (PFS) (i.e., the time form ILC diagnosis to a diagnosis of distant metastases or death, whichever occurred first), and pCR (German criteria, i.e., no invasive or *in situ* disease in the breast and axilla).

The categorical data were presented using n (%) and analyzed using the chi-squared test or Fisher’s exact test. The continuous data with a normal distribution (according to the Kolmogorov-Smirnov test) were presented as means ± standard deviations and analyzed using Student’s t-test. The continuous data with a skewed distribution were presented as medians (interquartile ranges (IQR)) and analyzed using the Mann-Whitney U-test. The Kaplan-Meier method was used to evaluate survival, and the curves were compared using the log-rank test. A Cox analysis was used to observe a correlation between the type of chemotherapy and the recurrence of breast cancer. The Cox analysis was also used to identify characteristics and risk factors for cancer recurrence and cofounding variables. The following variables were included in a multivariable analysis to adjust the survival analyses: age >55, T-stage, N-stage, surgery type, radiotherapy, and hormonal therapy. A selection process was made to eliminate non-significant covariables. Two-sided P-values ≤0.05 were considered statistically significant.

## 3 Available evidence from the authors’ center

From 1998 to 2016, 265 women were treated for ILC at the Center and were eligible to this study. Among them, 72 patients received NAC and 193 received adjuvant chemotherapy. There were no significant differences between the two groups regarding age, BMI, smoking, menopausal status, and use of hormonal replacement therapy (**Table 1**). All patients with HER2-positive disease after 2005 received anti-HER2 therapy. There were no significant differences between groups between the proportions of patients who received a sequential anthracycline-taxane, anthracycline-based, taxane-based, or other chemotherapy regimen.

**Table 1:** Characteristics of patients with breast invasive lobular carcinoma treated with neoadjuvant or adjuvant chemotherapy.

Variables	Neoadjuvant (n=72)	Adjuvant (n=193)	P
Laterality			0.782
Left	35 (48.6%)	89 (46.1%)	
Right	37 (51.4%)	104 (53.9%)	
Age (years)	57.0 ± 10.6	59.1 ± 9.4	0.118
Body mass index (kg/m^2^)	27.1 ± 5.1	26.5 ± 5.7	0.431
Smoking (ever)	40 (55.6%)	100 (51.8%)	0.678
Menopausal	45 (62.5%)	145 (75.1%)	
Hormonal replacement therapy	25 (34.7%)	70 (36.3%)	
T			<0.0001
1	11 (15.3%)	56 (29.0%)	
2	27 (37.5%)	101 (52.3%)	
3	13 (18.1%)	36 (18.7%)	
4	21 (29.2%)	0	
N			0.011
0	34 (47.2%)	68 (35.2%)	
1	34 (47.2%)	82 (42.5%)	
2	3 (4.2%)	18 (9.3%)	
3	1 (1.4%)	25 (13.0%)	
Estrogen receptors			0.784
Positive	67 (93.1%)	181 (93.8%)	
Negative	5 (6.9%)	12 (6.2%)	
Progesterone receptors			0.871
Positive	56 (77.8%)	147 (76.2%)	
Negative	16 (22.2%)	46 (23.8%)	
HER2-positive	5 (6.9%)	14 (7.3%)	>0.999
No cancer in breast AND lymph nodes after NAC	3 (4.2%)	–	–
Radiotherapy	60 (83.3%)	163 (84.5%)	0.851
Hormonotherapy	61 (84.7%)	167 (86.5%)	0.694
Regimen			0.438
Sequential anthracycline-taxane	35 (48.6%)	108 (56.0%)	
Anthracycline-based	15 (34.7%)	48 (24.9%)	
Taxane-based	16 (22.2%)	34 (17.6%)	
Others	6 (8.3%)	30 (15.5%)	
Mean follow-up (years)	8.0 ± 2.2	8.0 ± 4.2	0.960
Locoregional recurrence only			0.290
5-year	90.6%	99.5%	
10-year	90.6%	93.5%	
RFS			0.002
5-year	70.7%	86.9%	
10-year	51.8%	72.7%	
PFS			<0.0001
5-year	70.7%	87.8%	
10-year	59.3%	82.0%	
Death only			0.006
5-year	77.6%	91.6%	
10-year	56.2%	80.7%	

NAC, neoadjuvant chemotherapy; RFS, recurrence-free survival (local recurrence, distant metastasis, or death, whichever occurred first); PFS, progression-free survival (distant metastasis or death, whichever occurred first).

Significant differences in the initial staging of the disease were observed between the two groups. In the NAC group, patients were mostly stage 2a (31.9%) and 3b (29.2%), while the adjuvant group were mostly staged as 2a (26.4%) and 2b (30.1%). The patients with NAC and adjuvant chemotherapy patients were mainly T2 (37.5% and 52.3%, respectively), but almost 30% of the patients with NAC were T4 (29.2%) at diagnosis (**Table 1**).

In the NAC group, invasive carcinoma was found in the surgical specimen of 93.1% of the patients, and 68.1% of the patients had lymph node macrometastases. Therefore, only 4.2% of the patients with ILC achieved a pCR after NAC.

**Figure 1:**
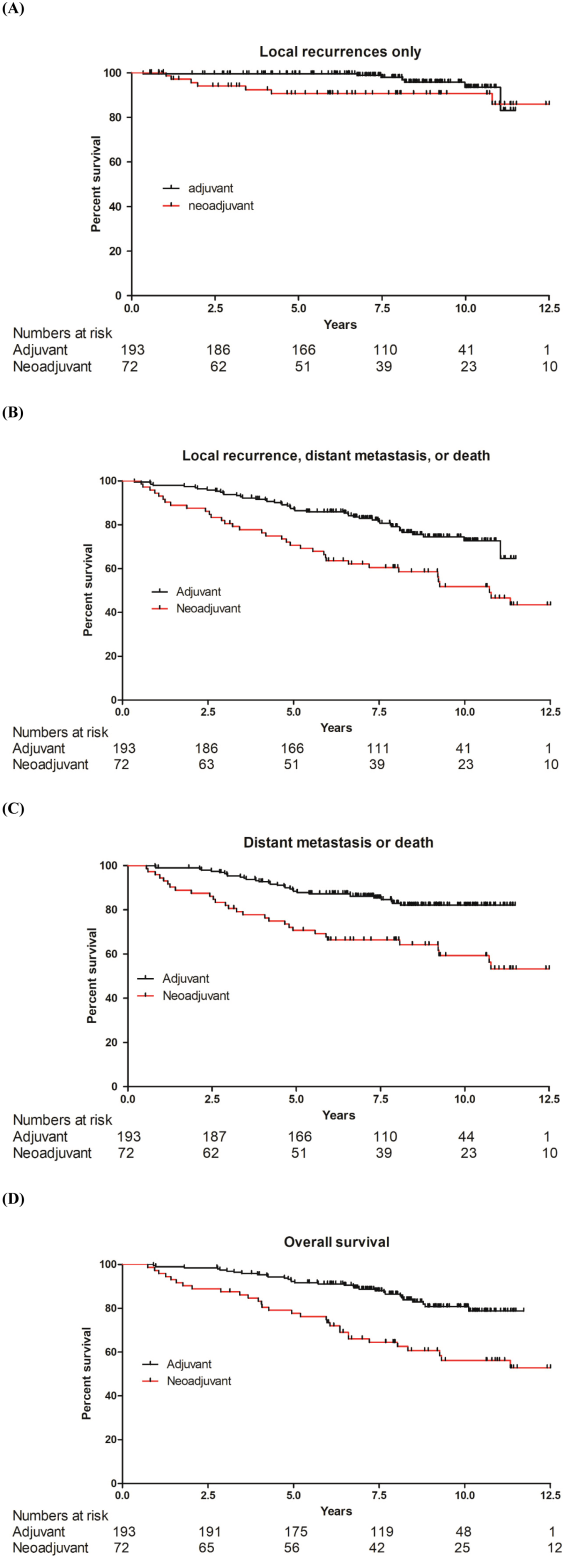
Kaplan-Meier analyses of survival in patients with lobular carcinoma of the breast treated with adjuvant vs. neoadjuvant chemotherapy. **(A)** Local recurrence. **(B)** Composite of local recurrence, distant metastases, or death, whichever occurred first. **(C)** Composite of distant metastases or death, whichever occurred first. **(D)** Overall survival.

The median follow-up was 8 years. The survival analyses were adjusted for age >55, T-stage, N-stage, surgery type, radiotherapy, and hormonal therapy. The two groups had a similar 10-year LRR (NAC: 90.6%; adjuvant: 93.5%, P=0.110). Compared with the adjuvant group, the NAC group showed a lower 10-year RFS (51.8% vs. 72.7%, P=0.0004), 10-year PFS (59.3% vs. 82.0%, P<0.0001), and 10-year OS (56.2% vs. 80.7%, P<0.0001) (**Table 1**, **Figure 1**).

## 4 Discussion and perspectives

This retrospective cohort study aimed to evaluate the survival parameters of patients with ILC after NAC vs. adjuvant chemotherapy. The results suggest that the response to NAC appears to be poorer than to adjuvant chemotherapy in patients with ILC after adjustment for confounders. These results could help guide the management of patients with ILC.

Retrospective data suggest adjuvant chemotherapy improves DFS and OS in early-stage ILC compared with no chemotherapy (13.3 vs. 7.5 years). However, this benefit diminishes when adjusted for stage (19). In the present study, NAC for ILC did not have any survival benefit over adjuvant therapy. These results are in line with a recent systematic review by Davey et al. (20) that included 28,000 patients; they concluded in no survival advantage in prescribing systemic chemotherapy (either adjuvant chemotherapy or NAC) in localized ILC, with a mean 10 years RFS of 75% in the two groups (20). In the present study, no differences were observed between groups regarding locoregional recurrence, as supported by Boughey et al. (21), but advantages of adjuvant therapy over NAC were seen for DFS, PFS, and OS. Of note, there were significant differences in patient characteristics between the two groups that could contribute to the differences in survival between the NAC and adjuvant groups. It is why the survival analyses were adjusted for age >55, T-stage, N-stage, surgery type, radiotherapy, and hormonal therapy. The differences in DFS, PFS, and OS remained statistically significant after adjusting for confounders. Nevertheless, that adjustment was statistical, and the results should be validated in future trials. Such trials could also consider neoadjuvant endocrine therapy with CDK4/6 inhibitors. Of note, studies suggested that patients with ILC harboring aggressive features (e.g., HER2+ or high Oncotype DX recurrence scores) may derive greater benefit from adjuvant chemotherapy (22, 23).

The results strongly suggest the importance of weighting the risks and benefits of administrating NAC to patients diagnosed with ILC. While BCS is an interesting surgical approach for patients with breast cancer due to its clear advantages for reconstruction and patient perspective, it is important to select those patients wisely. A recent retrospective study by Mukhtar et al. (12) on 69,000 patients with ILC in the US National Cancer Database concluded that surgery should be the first line of treatment in ILC, supporting the present study. Still, the present study goes against a study by Fitzal et al. (14) in 65 patients with ILC and a mean follow-up of 53 months that supported that NAC allowed for a higher rate of BCS in ILC (14). Therefore, there is still ambiguity in the literature related to NAC in ILC. Still, it is important to consider that giving NAC to a tumor not likely to respond can cause disease progression, which can impact the long-term survival of the patients and overall still require an aggressive surgical approach.

The main goal of NAC in HR-positive breast cancer is to downstage the breast disease and the nodal burden to allow for BCS and a less aggressive approach to the axillary disease. NAC has been associated with lower rates of tumor downstaging, higher rates of positive tumor margins, and fewer BCS in ILC compared with IDC (1, 24). pCR has been associated with higher survival in patients with breast cancer (25). A recent meta-analysis by O’Connor et al. (1) studied the sensitivity of ILC and IDC to NAC and its impact on the surgical approach on more than 85,000 patients. ILC shows significantly lower pCR rates to NACT compared to IDC (7.4% vs. 22.1%) (1, 26). It demonstrated that patients with ILC were less likely to achieve a pCR of the axilla or the breast but also underwent fewer BCS and had more positive margins compared with IDC. It is attributed to ILC’s slow-growing, HR+ nature and diffuse growth pattern (24, 26). Thornton et al. (27) reported no significant differences between NAC and neoadjuvant endocrine therapy in patients with ILC. Unfortunately, the meta-analysis did not examine survival (1). In the present study, only 4% the patients who received NAC achieved a pCR. Therefore, it was not possible to examine the factors involved in pCR and the impact of pCR on survival because of the too small number of events.

Many studies hypothesized that ILC might have a lesser sensitivity to NAC because of the association of ILC with other factors of poor prognosis rather than purely because of its biological characteristics (4, 5, 8). In fact, it has been reported that ILC tumors with positive hormonal receptor (HR) and negative HER2/neu status were less likely to achieve a pCR after NAC (4, 5). Quirke et al. (28) reported that higher-grade ILCs had a poorer response to NAC than lower-grade ILC. Ramalingam et al. (29) reported that although premenopausal women were more likely to receive NAC than menopausal ones, NAC was not associated with higher BCS rates. Due to the small number of participants in various categories, this study could not examine the impact of HR or HER2 on the outcomes, or the changes in chemotherapy regimens over time.

The evidence presented above had limitations. A retrospective design is usually associated with a selection bias. In addition, the analyses were limited to the data available in the registry. The study was performed at a single institution, resulting in a small number of patients when compared to the available meta-analyses (1, 20, 25), but those meta-analyses did not examine survival. Furthermore, it would have been interesting to consider partial pathological responses that still could allow breast conserving surgery and its impact on the survival of these patients. Due to the wide variety of chemotherapy regimens during the study period, analyses based on the exact regimens were not possible, and they were categorized as sequential anthracycline-taxane, anthracycline-based, taxane-based, and others. All cases were discussed in tumor boards for regimen selection. Only the overall pCR status (based on the breast and axilla complete responses, *in situ* not allowed) was available, and the separate responses in the breast or axilla were not available, preventing a finer analysis of the patients without pCR based on residual disease in the breast and/or the axilla. Finally, this study did not include a control group. In fact, all included patients received chemotherapy either in the neoadjuvant or adjuvant setting. Therefore, we cannot clearly adjudicate for adjuvant chemotherapy not knowing if it has a survival benefit compared to the absence of chemotherapy.

In conclusion, despite the general belief that invasive lobular and ductal breast carcinoma should be treated similarly, the present study suggests that ILC seems to have a poorer response to NAC than to adjuvant chemotherapy. It is the authors’ opinion that ILC should be considered separately from IDC in clinical trials and guidelines, and that patients with ILC might benefit from a more aggressive surgical approach followed by adjuvant chemotherapy no matter the severity of the disease. Of course, long-term prospective comparative studies and clinical trials are necessary to confirm the hypothesis.

## Data Availability

The raw data supporting the conclusions of this article will be made available by the authors, without undue reservation.
